# Raising Awareness for the Replication Crisis in Clinical Psychology by Focusing on Inconsistencies in Psychotherapy Research: How Much Can We Rely on Published Findings from Efficacy Trials?

**DOI:** 10.3389/fpsyg.2018.00256

**Published:** 2018-02-28

**Authors:** Michael P. Hengartner

**Affiliations:** Department of Applied Psychology, Zurich University of Applied Sciences, Zurich, Switzerland

**Keywords:** replication, clinical psychology, psychotherapy research, publication bias, allegiance, efficacy, effectiveness, methodology

The replication crisis addresses a fundamental problem in psychological research. Reported associations are systematically inflated and many published results do not replicate, suggesting that the scientific psychological literature is replete with false-positive findings (Pashler and Harris, 2012; Yong, 2012; Aarts et al., 2015). Unfortunately, the replication crisis remained almost unanswered in clinical psychology until very recently. Leichsenring et al. (2017) and Tackett et al. (2017) are to be complimented on their comprehensive recommendations for clinical science replicability, as these two contributions were the first to address this important topic with respect to clinical psychology. Their arguments are persuasive and elaborate, but some controversial topics not detailed by these authors need to be addressed in order to provide a critical appraisal of our most heeded research findings. Therefore, in order to raise awareness for the replication crisis in clinical psychology, I will outline some specific issues underscoring that inconsistent and systematically biased research findings persistently compromise the yield of clinical research. For it I will elaborate on the efficacy of psychotherapy, which arguably is the most cited research topic within clinical psychology.

## Publication and reporting bias inflates efficacy

Concerning replicability in psychotherapy research, the main question to pose is: How much can we rely on the published evidence? To start with it needs to be acknowledged that the average efficacy of psychotherapy based on the scientific literature is systematically overestimated due to publication bias (Cuijpers et al., 2010a; Driessen et al., 2015; Cristea et al., 2017a). In accordance with findings from psychopharmacological research (e.g., Turner et al., 2008), studies with unfavorable treatment outcome are less likely to be published in the scientific literature. For instance, Driessen et al. (2015) found that 24% of all trials aimed at evaluating the efficacy of psychological treatments for major depression funded by the National Institutes of Health were not published, which led to a 25% reduction in the estimated efficacy of psychotherapy (g = 0.52 vs. g = 0.39 after consideration of unpublished trials). Similarly, focusing exclusively on the efficacy of cognitive-behavioral therapy (CBT) for adult major depression, Cuijpers et al. (2010a) reported a reduction of 37% in efficacy after adjustment for publication bias (*d* = 0.67 vs. *d* = 0.42 after imputation of unpublished trials). On the individual study-level, some researchers use selective outcome reporting to illegitimately present findings in an opportunistic way. Outcome reporting bias is very prevalent in clinical science and indicates that authors omit or change primary outcomes on basis of the results in order to avoid undesired findings (Dwan et al., 2008). For instance, Kirkham et al. (2010) showed that adjusting for outcome reporting bias reduced the primary treatment effect by 20% or more in 23% of all meta-analyses of clinical trials reviewed. They further state that 19% of meta-analyses with an initially significant result became non-significant after adjustment for reporting bias. To the best of my knowledge, reporting bias was not systematically tested in psychotherapy research yet, but given its high prevalence in clinical science (Dwan et al., 2008) it is very likely that controlling for reporting bias would reduce the average efficacy of psychotherapy even further than sole correction for publication bias. Obtaining unbiased efficacy estimates for psychotherapy trials from the published literature is obviously a serious issue.

## Inconsistent meta-analyses

The replication crisis in the clinical sciences becomes also evident when one scrutinizes the literature on the comparative efficacy of different psychotherapies. The allegiance bias means that outcome studies in psychotherapy research are biased toward the main authors' psychotherapeutic allegiance (Luborsky et al., 1999). In this regard it is important to specifically mention three recent meta-analyses that came to completely divergent conclusions on the relative efficacy of CBT vs. psychodynamic therapy. In their meta-analysis, Leichsenring and Rabung (2011), both devoted to psychoanalysis, concluded that long-term psychodynamic therapy is markedly superior to short-term modalities such as CBT. Conversely, Smit et al. (2012), found no evidence for the superiority of long-term psychoanalysis related to their primary outcome of recovery as well as to all of their secondary outcomes comprising target problems, general psychiatric symptoms, personality pathology, social functioning, overall effectiveness, and quality of life. Finally, a meta-analysis conducted by Tolin (2010) concluded that CBT was superior to (short-term) psychodynamic therapy for depression and anxiety disorders. Obviously, and in accordance with an alarming issue recently detailed by Ferguson and Heene (2012), changes in the study selection criteria and the analysis procedure allow for producing almost any desired meta-analytic outcome. Unfortunately, the scientific literature is amassed with such examples. Thus, what shall we tell our patients: is long-term psychoanalysis empirically supported or would they fare better (or at least as good) with a short-term therapy such as CBT? However, that may be, clinicians and researcher should be aware that the credibility of many meta-analyses is rather modest (Pereira and Ioannidis, 2011).

## Systematic biases are pervasive

Another perennial hot topic in clinical psychology is the efficacy of pharmacological vs. psychological treatments. In a meta-analysis of direct comparisons, Cuijpers et al. (2013) as well as Huhn et al. (2014) found no significant differences between treatment modalities for panic disorder, generalized anxiety disorder and social phobia. Conversely, focusing on pre-post effect sizes, Bandelow et al. (2015) estimated that pharmacotherapy was largely superior to psychotherapy for these major anxiety disorders (*d* = 2.02, 95%-CI = 1.90–2.15, for medications vs. *d* = 1.22, 95%-CI = 1.14–1.30, for psychotherapies, *p* < 0.001). According to the authors this finding cannot be explained by heterogeneity, publication bias or allegiance effects (Bandelow et al., 2015). So, again a largely inconsistent finding impedes stringent clinical recommendations. Shall we recommend psychotropic drugs as first-line treatment for major anxiety disorders or is psychotherapy equally efficient? And what are the reasons for such striking discrepancies between aggregated study results? Cristea et al. (2017b) provide a partial explanation. In their recent meta-analysis they showed that trials who were funded by the pharmaceutical industry report slightly better outcomes for pharmacotherapy relative to psychotherapy. Indeed, research sponsored by the pharmaceutical industry or conducted by authors with industry-related financial conflicts of interest is systematically biased toward the industry's vested interests (Bekelman et al., 2003; Lexchin et al., 2003; Lundh et al., 2012). Apparently researchers can willingly produce results that match their (or their sponsors) expectations through questionable research practices (Simmons et al., 2011; Bakker et al., 2012). But financial interests and allegiance are only part of the story; reputation and promotion are equally powerful motives (Nosek et al., 2012). Differences in the study design are another explanation for inconsistencies between research findings. Khan et al. (2012) as well as Hróbjartsson et al. (2013) showed that unblinded trial assessors systematically overestimate the efficacy of the experimental intervention, and compared to pharmacotherapy trials, psychotherapy trials use significantly less blinded outcome assessors (Huhn et al., 2014). Given that participants in psychotherapy trials are not blinded, patients' treatment expectations and beliefs (see Chen et al., 2011) may further inflate the apparent efficacy of psychotherapeutic interventions. Finally, most psychotherapy trials use waitlist conditions as comparator. However, waitlist designs do not only produce larger efficacy estimates than trials with placebo or routine care comparator (Cuijpers et al., 2016), they may even impede or postpone spontaneous remission (Furukawa et al., 2014), which is referred to as a nocebo effect. The meta-analysis by Furukawa et al. (2014) is particularly revealing, as it showed that response rate in CBT for depression did not appreciably differ from psychological placebo (OR = 1.7), but it did so weakly from no-treatment conditions (OR = 2.4) and markedly from waitlist conditions (OR = 6.3). Likewise, comparing the effect of psychotherapy for major depression to pill placebo, Cuijpers et al. (2014b) found a poor effect size of g = 0.25, which is much smaller than the large effect sizes commonly obtained relative to waitlist conditions.

## On trial quality and effectiveness

Study quality is an important determinant of treatment efficacy in clinical science, but unfortunately, most published psychotherapy trials use poor methods such as small sample sizes, inadequate concealment of allocation, no intent-to-treat analyses, and unblinded outcome assessors (e.g., Newby et al., 2015; Cristea et al., 2017a). That hypothesis was stringently tested by Cuijpers et al. (2010b) with respect to psychotherapy for adult depression. Their results indeed revealed that high-quality studies are a small minority and that they yield remarkably lower mean effect size estimates than studies of lower quality (*d* = 0.22 vs. *d* = 0.74, *p* < 0.001). Using a continuous measure of study quality ranging from 0 to 8 points in a meta-regression showed that each additional point increase in study quality reduced the average effect size by −0.07 points (95%-CI = −0.09 to −0.05, *p* < 0.001). The impact of low-quality study bias was very recently replicated by Cristea et al. (2017a) in a meta-analysis of the efficacy of psychotherapy for borderline personality disorder, suggesting that these findings are generalizable. Worthy of note, the estimates outlined above refer almost exclusively to efficacy under controlled laboratory conditions using selected, unrepresentative patient samples. Just as in pharmacological research (see Naci and Ioannidis, 2015), evidence of efficacy for psychological interventions under optimal laboratory conditions often does not replicate in real world clinical settings (Westen et al., 2004). Due to selective samples and unrepresentative clinical settings, effectiveness of many empirically-supported psychological interventions is inadequately poor under naturalistic real-world conditions (Weisz et al., 1995; Hansen et al., 2002; Westen et al., 2004). Furthermore, some psychological interventions with proven laboratory-based efficacy turned out largely ineffective (Hallfors and Cho, 2007) or even harmful (Lilienfeld, 2007) in real-world effectiveness trials. That is, efficacy estimates are not only inflated due to scientific and methodological biases, they also poorly translate into measurable public health benefits. However, a crucial point to consider is: “What do psychotherapy trials actually measure?” Following the primacy of the biomedical model of mental disorder, clinical psychology has largely adapted the methods from pharmacology trials (Deacon, 2013). That is, symptom rating scales have become the primary outcome in most trials, but this is not necessarily the domain where psychotherapy has its most significant impact. Perhaps psychotherapy's major asset, in contrast to pharmacological treatments, is to improve social functioning (e.g., Fournier et al., 2015). Replicating effectiveness within these domains is perhaps even more challenging than replicating symptom-based efficacy.

## Summary and conclusions

As in other psychological specialties (see Bakker et al., 2012), effect sizes published in the clinical psychological literature are often heterogeneous and inflated due to various scientific biases including allegiance bias (Luborsky et al., 1999), publication bias (Driessen et al., 2015), unblinded outcome assessors (Khan et al., 2012), sponsorship bias (Cristea et al., 2017b), or small sample sizes (Cuijpers et al., 2010b). After adjustment for systematic biases, efficacy estimates for various psychotherapy modalities tend to be disappointingly small (Cuijpers et al., 2010b; Cristea et al., 2017a). Some evidence suggests that when efficacy is estimated based exclusively on unbiased high-quality trials, effects of psychotherapy could fall below the threshold for clinical relevance (Cuijpers et al., 2014a). Recently, some psychotherapy researchers hence raised the controversial point that effects of both psychotherapy and pharmacotherapy for depression may entirely reflect a placebo effect (Cuijpers and Cristea, 2015). Of further concern is the gap between treatment efficacy in controlled laboratory trials and treatment effectiveness in naturalistic real-world settings (Westen et al., 2004; Hallfors and Cho, 2007). The literature reviewed in this commentary was restricted to the efficacy of clinical psychological interventions, as that topic is highly relevant for clinical psychology. Nevertheless, conflicting and irreproducible findings have been detected and discussed in various other hot topics within clinical psychology, including the debatable effect of menopause on the occurrence of depression (Rössler et al., 2016; Hengartner, 2017), the putative consequences of violent video games (Ferguson and Kilburn, 2010; Calvert et al., 2017), or inconsistent associations between psychopathology and stress physiology (Chida and Hamer, 2008; Rosmalen and Oldehinkel, 2011). Even though the replication crisis was mostly addressed within social psychology, I conclude that it is no less pernicious and prevalent in clinical psychology. Psychotherapy was a marvelous invention, but initial enthusiasm regarding its efficacy has now been obfuscated due to scientific biases that systematically inflate estimates. Being aware of these issues may certainly improve our scientific and clinical endeavors.

## Author contributions

The author confirms being the sole contributor of this work and approved it for publication.

### Conflict of interest statement

The author declares that the research was conducted in the absence of any commercial or financial relationships that could be construed as a potential conflict of interest.

## References

[B1] Aarts A. A. Anderson J. E. Anderson C. J. Attridge P. R. Attwood A. Axt J. (2015). Estimating the reproducibility of psychological science. Science 349:aac4716 10.1126/science.aac4716 26315443

[B2] Bakker M. van Dijk A. Wicherts J. M. (2012). The rules of the game called psychological science. Perspect. Psychol. Sci. 7, 543–554. 10.1177/1745691612459060 26168111

[B3] Bandelow B. Reitt M. Röver C. Michaelis S. Görlich Y. Wedekind D. (2015). Efficacy of treatments for anxiety disorders: a meta-analysis. Int. Clin. Psychopharmacol. 30, 183–192. 10.1097/YIC.0000000000000078 25932596

[B4] Bekelman J. E. Li Y. Gross C. P. (2003). Scope and impact of financial conflicts of interest in biomedical research: a systematic review. JAMA 289, 454–465. 10.1001/jama.289.4.454 12533125

[B5] Calvert S. L. Appelbaum M. Dodge K. A. Graham S. Nagayama Hall G. C. Hamby S.. (2017). The american psychological association task force assessment of violent video games: science in the service of public interest. Am. Psychol. 72, 126–143. 10.1037/a0040413 28221065

[B6] Chen J. A. Papakostas G. I. Youn S. J. Baer L. Clain A. J. Fava M.. (2011). Association between patient beliefs regarding assigned treatment and clinical response: reanalysis of data from the Hypericum Depression Trial Study Group. J. Clin. Psychiatry 72, 1669–1676. 10.4088/JCP.10m06453 22053942

[B7] Chida Y. Hamer M. (2008). Chronic psychosocial factors and acute physiological responses to laboratory-induced stress in healthy populations: a quantitative review of 30 years of investigations. Psychol. Bull. 134, 829–885. 10.1037/a0013342 18954159

[B8] Cristea I. A. Gentili C. Cotet C. D. Palomba D. Barbui C. Cuijpers P. (2017a). Efficacy of psychotherapies for borderline personality disorder: a systematic review and meta-analysis. JAMA Psychiatry 74, 319–328. 10.1001/jamapsychiatry.2016.4287 28249086

[B9] Cristea I. A. Gentili C. Pietrini P. Cuijpers P. (2017b). Sponsorship bias in the comparative efficacy of psychotherapy and pharmacotherapy for adult depression: meta-analysis. Br. J. Psychiatry 210, 16–23. 10.1192/bjp.bp.115.179275 27810891

[B10] Cuijpers P. Cristea I. A. (2015). What if a placebo effect explained all the activity of depression treatments? World Psychiatry 14, 310–311. 10.1002/wps.20249 26407786 PMC4592653

[B11] Cuijpers P. Cristea I. A. Karyotaki E. Reijnders M. Huibers M. J. (2016). How effective are cognitive behavior therapies for major depression and anxiety disorders? A meta-analytic update of the evidence. World Psychiatry 15, 245–258. 10.1002/wps.20346 27717254 PMC5032489

[B12] Cuijpers P. Sijbrandij M. Koole S. L. Andersson G. Beekman A. T. Reynolds C. F. III. (2013). The efficacy of psychotherapy and pharmacotherapy in treating depressive and anxiety disorders: a meta-analysis of direct comparisons. World Psychiatry 12, 137–148. 10.1002/wps.20038 23737423 PMC3683266

[B13] Cuijpers P. Smit F. Bohlmeijer E. Hollon S. D. Andersson G. (2010a). Efficacy of cognitive-behavioural therapy and other psychological treatments for adult depression: meta-analytic study of publication bias. Br. J. Psychiatry 196, 173–178. 10.1192/bjp.bp.109.066001 20194536

[B14] Cuijpers P. Turner E. H. Koole S. L. van Dijke A. Smit F. (2014a). What is the threshold for a clinically relevant effect? The case of major depressive disorders. Depress. Anxiety 31, 374–378. 10.1002/da.22249 24677535

[B15] Cuijpers P. Turner E. H. Mohr D. C. Hofmann S. G. Andersson G. Berking M.. (2014b). Comparison of psychotherapies for adult depression to pill placebo control groups: a meta-analysis. Psychol. Med. 44, 685–695. 10.1017/S0033291713000457 23552610

[B16] Cuijpers P. van Straten A. Bohlmeijer E. Hollon S. D. Andersson G. (2010b). The effects of psychotherapy for adult depression are overestimated: a meta-analysis of study quality and effect size. Psychol. Med. 40, 211–223. 10.1017/S0033291709006114 19490745

[B17] Deacon B. J. (2013). The biomedical model of mental disorder: a critical analysis of its validity, utility, and effects on psychotherapy research. Clin. Psychol. Rev. 33, 846–861. 10.1016/j.cpr.2012.09.007 23664634

[B18] Driessen E. Hollon S. D. Bockting C. L. Cuijpers P. Turner E. H. (2015). Does publication bias inflate the apparent efficacy of psychological treatment for major depressive disorder? a systematic review and meta-analysis of us national institutes of health-funded trials. PLoS ONE 10:e0137864. 10.1371/journal.pone.0137864 26422604 PMC4589340

[B19] Dwan K. Altman D. G. Arnaiz J. A. Bloom J. Chan A. W. Cronin E.. (2008). Systematic review of the empirical evidence of study publication bias and outcome reporting bias. PLoS ONE 3:e3081. 10.1371/journal.pone.0003081 18769481 PMC2518111

[B20] Ferguson C. J. Heene M. (2012). A Vast Graveyard of undead theories: publication bias and psychological science's aversion to the null. Perspect. Psychol. Sci. 7, 555–561. 10.1177/1745691612459059 26168112

[B21] Ferguson C. J. Kilburn J. (2010). Much ado about nothing: the misestimation and overinterpretation of violent video game effects in eastern and western nations: comment on Anderson et al. (2010). Psychol. Bull. 136, 174–178. 10.1037/a0018566 20192554

[B22] Fournier J. C. DeRubeis R. J. Amsterdam J. Shelton R. C. Hollon S. D. (2015). Gains in employment status following antidepressant medication or cognitive therapy for depression. Br. J. Psychiatry 206, 332–338. 10.1192/bjp.bp.113.133694 24925985 PMC4264990

[B23] Furukawa T. A. Noma H. Caldwell D. M. Honyashiki M. Shinohara K. Imai H.. (2014). Waiting list may be a nocebo condition in psychotherapy trials: a contribution from network meta-analysis. Acta Psychiatr. Scand. 130, 181–192. 10.1111/acps.12275 24697518

[B24] Hallfors D. Cho H. (2007). Moving behavioral science from efficacy to effectiveness. Int. J. Behav. Consult. Ther. 3, 236–250. 10.1037/h0100801

[B25] Hansen N. B. Lambert M. J. Forman E. M. (2002). The psychotherapy dose-response effect and its implications for treatment delivery services. Clin. Psychol. Sci. Pract. 9, 329–343. 10.1093/clipsy.9.3.329

[B26] Hengartner M. P. (2017). Subtle scientific fallacies undermine the validity of neuroendocrinological research: do not draw premature conclusions on the role of female sex hormones. Front. Behav. Neurosci. 11:3 10.3389/fnbeh.2017.00003 28144217 PMC5239782

[B27] Hróbjartsson A. Thomsen A. S. Emanuelsson F. Tendal B. Hilden J. Boutron I.. (2013). Observer bias in randomized clinical trials with measurement scale outcomes: a systematic review of trials with both blinded and nonblinded assessors. CMAJ 185, E201–211. 10.1503/cmaj.120744 23359047 PMC3589328

[B28] Huhn M. Tardy M. Spineli L. M. Kissling W. Förstl H. Pitschel-Walz G.. (2014). Efficacy of pharmacotherapy and psychotherapy for adult psychiatric disorders: a systematic overview of meta-analyses. JAMA Psychiatry 71, 706–715. 10.1001/jamapsychiatry.2014.112 24789675

[B29] Khan A. Faucett J. Lichtenberg P. Kirsch I. Brown W. A. (2012). A systematic review of comparative efficacy of treatments and controls for depression. PLoS ONE 7:e41778. 10.1371/journal.pone.0041778 22860015 PMC3408478

[B30] Kirkham J. J. Dwan K. M. Altman D. G. Gamble C. Dodd S. Smyth R.. (2010). The impact of outcome reporting bias in randomised controlled trials on a cohort of systematic reviews. BMJ 340:c365. 10.1136/bmj.c365 20156912

[B31] Leichsenring F. Abbass A. Hilsenroth M. J. Leweke F. Luyten P. Keefe J. R.. (2017). Biases in research: risk factors for non-replicability in psychotherapy and pharmacotherapy research. Psychol. Med. 47, 1000–1011. 10.1017/S003329171600324X 27955715

[B32] Leichsenring F. Rabung S. (2011). Long-term psychodynamic psychotherapy in complex mental disorders: update of a meta-analysis. Br. J. Psychiatry 199, 15–22. 10.1192/bjp.bp.110.082776 21719877

[B33] Lexchin J. Bero L. A. Djulbegovic B. Clark O. (2003). Pharmaceutical industry sponsorship and research outcome and quality: systematic review. BMJ 326, 1167–1170. 10.1136/bmj.326.7400.1167 12775614 PMC156458

[B34] Lilienfeld S. O. (2007). Psychological treatments that cause harm. Perspect. Psychol. Sci. 2, 53–70. 10.1111/j.1745-6916.2007.00029.x 26151919

[B35] Luborsky L. Diguer L. Seligman D. A. Rosenthal R. Krause E. D. Johnson S. (1999). The researcher's own therapy allegiances: a “wild card” in comparisons of treatment efficacy. Clin. Psychol. Sci. Pract. 6, 95–106. 10.1093/clipsy/6.1.95

[B36] Lundh A. Sismondo S. Lexchin J. Busuioc O. A. Bero L. (2012). Industry sponsorship and research outcome. Cochrane Database Syst. Rev. 12:MR000033. 10.1002/14651858.MR000033.pub2 23235689

[B37] Naci H. Ioannidis J. P. (2015). How good is “evidence” from clinical studies of drug effects and why might such evidence fail in the prediction of the clinical utility of drugs? Annu. Rev. Pharmacol. Toxicol. 55, 169–189. 10.1146/annurev-pharmtox-010814-124614 25149917

[B38] Newby J. M. McKinnon A. Kuyken W. Gilbody S. Dalgleish T. (2015). Systematic review and meta-analysis of transdiagnostic psychological treatments for anxiety and depressive disorders in adulthood. Clin. Psychol. Rev. 40, 91–110. 10.1016/j.cpr.2015.06.002 26094079

[B39] Nosek B. A. Spies J. R. Motyl M. (2012). Scientific Utopia: II. restructuring incentives and practices to promote truth over publishability. Perspect. Psychol. Sci. 7, 615–631. 10.1177/1745691612459058 26168121 PMC10540222

[B40] Pashler H. Harris C. R. (2012). Is the replicability crisis overblown? three arguments examined. Perspect. Psychol. Sci. 7, 531–536. 10.1177/1745691612463401 26168109

[B41] Pereira T. V. Ioannidis J. P. (2011). Statistically significant meta-analyses of clinical trials have modest credibility and inflated effects. J. Clin. Epidemiol. 64, 1060–1069. 10.1016/j.jclinepi.2010.12.012 21454050

[B42] Rosmalen J. G. Oldehinkel A. J. (2011). The role of group dynamics in scientific inconsistencies: a case study of a research consortium. PLoS Med. 8:e1001143. 10.1371/journal.pmed.1001143 22180733 PMC3236735

[B43] Rössler W. Ajdacic-Gross V. Riecher-Rossler A. Angst J. Hengartner M. P. (2016). Does menopausal transition really influence mental health? Findings from the prospective long-term Zurich study. World Psychiatry 15, 146–154. 10.1002/wps.20319 27265705 PMC4911775

[B44] Simmons J. P. Nelson L. D. Simonsohn U. (2011). False-positive psychology: undisclosed flexibility in data collection and analysis allows presenting anything as significant. Psychol. Sci. 22, 1359–1366. 10.1177/0956797611417632 22006061

[B45] Smit Y. Huibers M. J. Ioannidis J. P. van Dyck R. van Tilburg W. Arntz A. (2012). The effectiveness of long-term psychoanalytic psychotherapy–a meta-analysis of randomized controlled trials. Clin. Psychol. Rev. 32, 81–92. 10.1016/j.cpr.2011.11.003 22227111

[B46] Tackett J. L. Lilienfeld S. O. Patrick C. J. Johnson S. L. Krueger R. F. Miller J. D.. (2017). It's time to broaden the replicability conversation: thoughts for and from clinical psychological science. Perspect. Psychol. Sci. 12, 742–756. 10.1177/1745691617690042 28972844

[B47] Tolin D. F. (2010). Is cognitive-behavioral therapy more effective than other therapies? A meta-analytic review. Clin. Psychol. Rev. 30, 710–720. 10.1016/j.cpr.2010.05.003 20547435

[B48] Turner E. H. Matthews A. M. Linardatos E. Tell R. A. Rosenthal R. (2008). Selective publication of antidepressant trials and its influence on apparent efficacy. N. Engl. J. Med. 358, 252–260. 10.1056/NEJMsa065779 18199864

[B49] Weisz J. R. Donenberg G. R. Han S. S. Weiss B. (1995). Bridging the gap between laboratory and clinic in child and adolescent psychotherapy. J. Consult. Clin. Psychol. 63, 688–701. 10.1037/0022-006X.63.5.688 7593861

[B50] Westen D. Novotny C. M. Thompson-Brenner H. (2004). The empirical status of empirically supported psychotherapies: assumptions, findings, and reporting in controlled clinical trials. Psychol. Bull. 130, 631–663. 10.1037/0033-2909.130.4.631 15250817

[B51] Yong E. (2012). Replication studies: bad copy. Nature 485, 298–300. 10.1038/485298a 22596136

